# An Engineered Heat-Inducible Expression System for the Production of Casbene in *Nicotiana benthamiana*

**DOI:** 10.3390/ijms241411425

**Published:** 2023-07-13

**Authors:** Edith C. F. Forestier, Amy C. Cording, Gary J. Loake, Ian A. Graham

**Affiliations:** 1 Centre for Novel Agricultural Products (CNAP), Department of Biology, University of York, Wentworth Way, York YO10 5DD, UK; ef462@cam.ac.uk (E.C.F.F.);; 2 Institute of Molecular Plant Sciences, Daniel Rutherford Building, School of Biological Sciences, University of Edinburgh, Kings Buildings, Mayfield Road, Edinburgh EH9 3JH, UK; g.loake@ed.ac.uk

**Keywords:** *Agrobacterium tumefaciens*, casbene, diterpenes, heat-shock, MoClo, *Nicotiana benthamiana*, promoters, stable transformation

## Abstract

Plants respond to heat stress by producing heat-shock proteins. These are regulated by heat-shock promoters containing regulatory elements, which can be harnessed to control protein expression both temporally and spatially. In this study, we designed heat-inducible promoters to produce the diterpene casbene in *Nicotiana benthamiana*, through a multi-step metabolic pathway. To potentially increase gene transcription, we coupled heat-shock elements from *Arabidopsis thaliana* Hsp101 or *Glycine max* GmHsp17.3-B promoters, CAAT and TATA boxes from CaMV *35S*, and the 5′UTR from the tobacco mosaic virus. The resulting four chimeric promoters fused to a green fluorescent protein (GFP) reporter showed that the variant Ara2 had the strongest fluorescent signal after heat shock. We next created a 4-gene cassette driven by the *Ara2* promoter to allow for exogenous synthesis of casbene and transformed this multigene construct along with a selectable marker gene into *Nicotiana benthamiana*. Metabolic analysis on the transgenic lines revealed that continuous heat outperforms heat shock, with up to 1 μg/mg DW of casbene detected after 32 h of uninterrupted 40 °C heat. These results demonstrate the potential of heat-inducible promoters as synthetic biology tools for metabolite production in plants.

## 1. Introduction

Discovery of the heat-shock response in the fly *Drosophila melanogaster* [1] led to the further finding of heat-shock proteins (HSP) twelve years later [2]. Additional studies subsequently revealed several groups of these proteins in all living organisms including plants [3]. The complex signal transduction response, following heat stress, results in the production of heat-related transcription factors, which in turn trigger the synthesis of heat-shock proteins [4]. The transcription factors interact with specific short DNA sequences defined as heat-shock elements (HSEs). These are usually multiple NGAAN sequences found in both orientations in the promoter region of heat-shock genes [5,6]. A challenge since then has been to exploit these heat-shock promoters as a tool for plant bioengineering, resulting in numerous proof-of-concept publications using reporter genes [7,8,9]. Practical applications appear to be more advanced in *Escherichia coli* [10] as one research group has successfully produced natural products such as curcumin and hydroxycinnamic acids using heat-shock promoters [11].

Our research focused on the production of the gem-dimethylcyclopropyl class of bioactive casbene-derived diterpenoids from *Euphorbiaceae* species, using *Nicotiana benthamiana* as a plant platform to synthesize these exogenous molecules [12]. Tiglianol tigliate is for example a casbene-derived veterinary pharmaceutical, registered to treat mast cell tumors in dogs [13]. However, it is currently only extracted from *Fontainea picrosperma* seeds, a sub-canopy tree native to a restricted area of the Queensland rainforest [14]. Tiglianol tigliate is presumed to be synthesized from casbene through jolkinol C [15,16,17], making both compounds compelling targets to accumulate in plants for potential semi-synthesis applications. In a previous work, we successfully obtained constitutive transgenic lines producing jolkinols—albeit with a phenotype and delayed growth [12]—but we failed to generate transgenic constitutive lines accumulating casbene. Hence, we explored the use of inducible promoters to conditionally express the cognate biosynthetic genes in an attempt to produce casbene without affecting plant growth and fitness. The rationale behind employing inducible promoters is to facilitate healthy and normal growth of the plants under optimal growth conditions until they attain an ideal size and biomass. We can minimize toxicity effects during normal growth, as the elevated levels of target proteins or metabolites are only produced during the induction phase. This approach stands in contrast to constitutive expression, which may potentially lead to adverse toxic effects.

Even though a typical T-DNA size is between 10 and 30 kb [18], we utilized short promoter sequences to reduce the T-DNA size as much as possible in order to increase the chances of successful stable transformation. We avoided using any chemically induced systems, such as the *Alc* regulon induced by ethanol [19], the pOp/LhGR system induced by dexamethasone [20], or the LexA/XVE complex induced by estradiol [21]. These combinations would increase the construct’s size considerably because the gene encoding the chemical-reacting transcription factor, subsequently binding to the associated promoter to initiate transcription, must also be integrated. We focused on heat-shock promoter sequences, relying on the endogenous pool of transcription factors in *N. benthamiana* that are produced after a thermal stress. We also excluded tissue-specific promoters, as we aimed to accumulate the product in the whole plant for maximum biomass. Our objective was to generate transgenic lines capable of attaining a growth rate approximately equivalent to that of the wild-type plants. Subsequently, metabolite production would be induced once the plants have achieved adequate growth. It is important to note that at this stage, the primary focus is on the production of desired metabolites, and the well-being of the plants is not a primary concern as they will be ultimately sacrificed for harvest. 

Dissection of the heat-shock promoter domains of soybean *Gmhsp17* genes identified multiple regulatory elements, including the HSEs, together with CAAT and TATA boxes [22,23]. Subsequently, chimeric promoters were created by mixing these regulatory elements from different promoters of diverse species to form a single functional promoter [24,25]. We exploited this strategy to develop a set of synthetic promoters, which were then tested in *N. benthamiana* for casbene production.

## 2. Results

### 2.1. Design of Chimeric Heat-Shock Promoters

Our design was inspired by the successful approach of Schöffl and colleagues, who combined varying lengths of the *Glycine max* promoter Gmhsp17.3-B with the TATA box sequence of the CaMV 35S promoter [24]. We considered employing different portions of the 35S promoter region containing the TATA and CAAT boxes and in this regard were influenced by the work of Ow and co-workers, who studied the functional regions of the 35S promoter, dividing it into distal, medial, and proximal regions [26]. This approach was also adopted by other studies during the same period [27,28,29].

**Figure 1:**
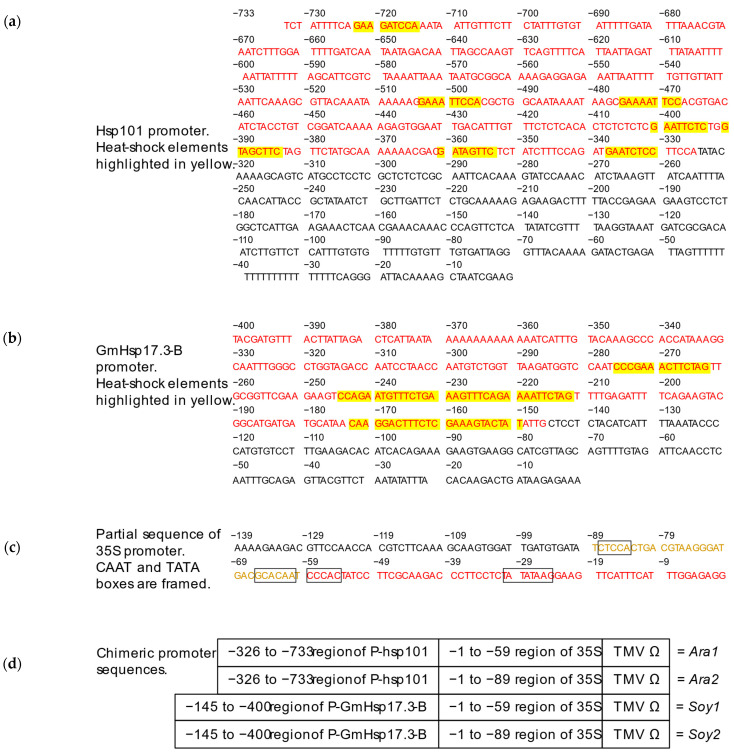
Design of the chimeric heat-shock promoters. (**a**) Whole native Hsp101 heat-shock promoter. (**b**) Whole native GmHsp17.3-B heat-shock promoter. (**c**) Partial sequence of the 35S promoter with framed CAAT and TATA boxes. (**d**) Chimeric promoters designed using the sequences displayed in red and orange from (**a**–**c**). TMV Ω plays the role of 5′UTR.

We selected the well-characterized heat-shock promoters GmHsp17.3B from *Glycine max* (P- Gmhsp17.3-B) and Hsps101 from *Arabidopsis thaliana* (P-Hsp101) [30] as the heat-shock elements within these promoters were already identified, allowing for the design of synthetic promoters targeting the heat-shock response. We combined a partial region of P-Hsp101 (Figure 1a) or P-GmHsp17.3-B (Figure 1b) covering all heat-shock elements, with different length sequences of the 35S promoter including only the medial and proximal parts (Figure 1c), to which we added the 5′UTR of tobacco mosaic virus (TMV) (Figure 1d). We took the entire medial and proximal region of the 35S promoter (−1 to −89) containing three CAAT boxes and one TATA box, which coincided with the −1 to −90 core promoter [31] (Figure 1c). We also tested a shorter 35S sequence containing only one CAAT and the TATA box (−1 to −59) (Figure 1c), which is longer than the minimal 35S promoter (−1 to −46) already used with transcription control systems to express transgenes in plants [20,32]. We added the 5′ leader of TMV from the MoClo Plant Parts kit [33], as it had been reported to increase the level of foreign gene transcripts in vitro and in vivo [34]. 

In our endeavor to clone and reconstitute the original promoters, we encountered challenges during the synthesis and sequencing process, particularly with regard to the polyT region in the Hsp101 5′UTR and the polyA region in the GmHsp17.3B 5′UTR. The Hsp101 5′UTR had to be reduced from 22-nucleotide to 12-nucleotides polyT, while the GmHsp17.3B 5′UTR was reduced from 14 to 13-nucleotides polyA. These modifications were made to facilitate synthesis and validate the sequencing results. Therefore, they will be referred to as native-like promoters. In addition to the two native-like promoters P- Hsp101 and P-GmHsp17.3-B, we created four chimeric promoters, Ara1, Ara2, Soy1, and Soy2 (Figure 1d), whose maximum size was 569 nucleotides. We also purchased two commercial promoters Hsp18.2 (P-Hsp18.2) [35] and Hsp70B (P-Hsp70B) [36] from *A. thaliana*, which were provided by the Goldenbraid synthetic biology kit [37]. Together we generated eight distinct promoters that we linked to turbo GFP (tGFP) and the OCS terminator from the MoClo kit [33,38,39].

### 2.2. Transient Expression Test of GFP Constructs Driven by Heat-Shock Promoters in Nicotiana benthamiana

**Figure 2:**
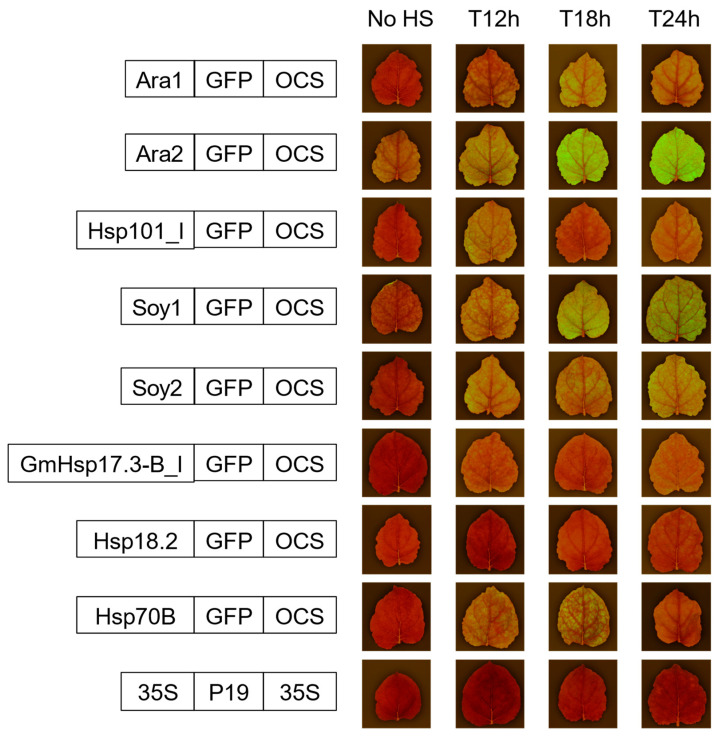
GFP expression driven by chimeric and original heat-shock promoters after transient expression in *N. benthamiana* leaves. Visualization of fluorescence at different time-points after 2 h of heat shock. The non-heat-shocked leaves (no HS) were all collected at time-point T12h.

We conducted transient expression assays in *N. benthamiana* using *Agrobacterium tumefaciens*-mediated infiltration with the eight GFP constructs mentioned above, followed four days later by a heat-shock treatment at 40 °C for 2 h. A study had previously shown that a 30 min heat-shock of 37 °C one or two days after agroinfiltration improved the production of the target protein [40]; we did not know, however, if our heating conditions could boost GFP production in the same way. Visual assessment of GFP expression under UV light was performed at various time points post heat-shock and provided valuable preliminary insights into promoter activity. At specific time points, the triplicate samples of plants expressing the same construct exhibited similar fluorescence patterns, therefore a representative leaf was carefully selected for photography (Figure 2 and Figure S1). However, the constructs with Hsp101-like and GmHsp17.3B-like promoters did not show a consistent pattern of fluorescence fluctuation over time, with levels stronger at T12h and T24h than T18h. Notably, among the eight tested promoters, the Ara2-tGFP-OCS construct exhibited remarkably stronger fluorescence, surpassing the others at T18h. This pronounced onset of fluorescence suggests the potential of this specific promoter to drive robust gene expression. 

Interestingly, these experiments also revealed that the efficiency of the synthetic promoters did not follow a predictable pattern. Indeed, the Ara2 promoter demonstrated superior performance compared to Ara1 while Soy1 outperformed Soy2 at T18h. These results suggest that the length of the 35S fragment alone may not significantly influence promoter efficiency. These findings highlight the complex interplay between promoter elements and their potential synergistic effects in driving gene expression.

Following the promising results obtained with the Ara2 promoter, we chose this promoter to express four genes for casbene production within a single vector and further, to stably transform the resulting construct into *N. benthamiana*.

### 2.3. Stable Transformation of a Multigene Construct Driven by the Ara2 Promoter and Analysis of Casbene Production in the Associated Transgenic Lines

**Figure 3:**
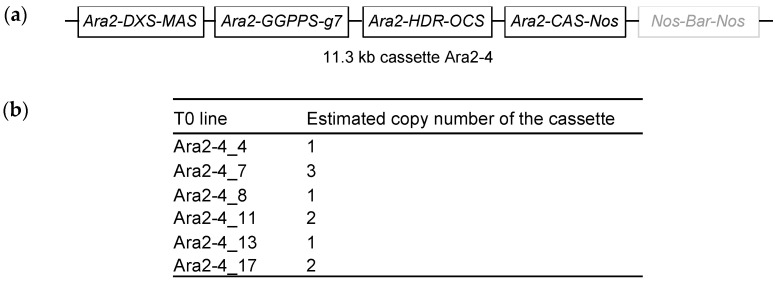
T-DNA construct design for stable transformation of heat-inducible expression of genes involved in the synthesis of casbene in *Nicotiana benthamiana*. (**a**) Multigene cassette for the stable expression in *N. benthamiana*. Precursor supply genes (*DXS*, *HDR*, and *GGPPS*) and *CAS* are under the control of chimeric Ara2 promoter. Basta resistance gene (*Bar*) is driven by a constitutive promoter provided by the MoClo kit. Cassette called Ara2-4 in reference to the 4 genes allowing the synthesis of casbene. (**b**) Viable primary transformants (T0) obtained after leaf disc transformation with Ara2-4 cassette and estimation of the copy number of integrated cassette based on segregation analysis.

We previously reported that in order to produce maximum exogenous casbene in *N. benthamiana*, certain genes of the MEP pathway—a plastid biosynthetic pathway that synthesizes diterpene precursors [41]—need to be overexpressed. We established that the genes responsible for the first and last step in the MEP pathway—1-deoxy-d-xylulose-5-phosphate synthase (*DXS*) and 4-hydroxy-3-methylbut-2-enyl diphosphate reductase (*HDR*), in combination with geranylgeranyl pyrophosphate synthase (*GGPPS*) and casbene synthase (*CAS*)—are optimal for increasing flux through to casbene in *N. benthamiana* [12]. We therefore cloned *DXS*, *HDR*, *GGPPS*, and *CAS* genes into a single MoClo vector driven by the Ara2 promoter for stable transformation. Because of the potential of sequence homology-dependent gene silencing [42], each of these four genes was placed under the control of a different transcriptional terminator given by the MoClo kit (Figure 3a, Table S1). We created a multigene construct labeled Ara2-4 to refer to the four genes involved in casbene synthesis; the vector also included a constitutively expressed Bar gene to convey Basta^®^ resistance following stable transformation (Figure 3a).

The 11.3 kb cassette was successfully introduced into *N. benthamiana*, resulting in the generation of 18 primary transformants (T0s) by random T-DNA integration, using *Agrobacterium*-mediated transformation [43]. However, only six of these primary transformants resulted in viable T1 plants (Figure 3b). We estimated the copy number of the transgene by segregation on Basta^®^ and identified three single copy T0s, Ara2-4 n°4, n°8, and n°13. We grew the T1 then T2 progenies to select homozygous lines and attained the T3 generation to perform metabolic analyses. No discernible phenotype was observed in the T3 transgenic lines based on external examination alone (Figure S2).

**Figure 4:**
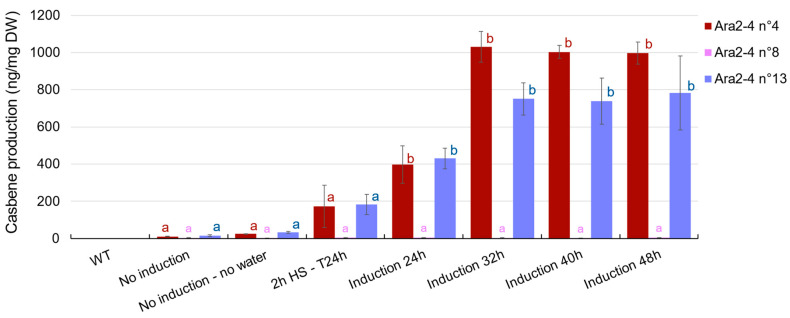
Casbene production after heat-shock or heat-induction in different transgenic lines Ara2- 4 (T3 generation). Average casbene production (in ng/mg DW) ± standard deviation (n = 3). Letters indicate the statistical differences between each heat exposure (*p* < 0.05, one-way ANOVA and Tukey’s multiple comparisons test). Each color represents an unrelated statistical group. “2 h HS T24h” means the plants were placed at 40 °C for 2 h then replaced in a normal environment to be harvested 24 h later. “Induction xxh” means the plants were in a continuous heat at 40 °C for xxh before being harvested. The non-induced plants were harvested at the same time as the plants induced for 48 h.

We determined the content of casbene in these T3 lines, initially by heat-shocking the plants as before, i.e., 40 °C for 2 h and then collecting the material after 24 h. We also tried heating the plants for a longer period and discovered that they could endure this heat without water for up to 32 h before showing signs of distress. Interestingly, casbene accumulated in the plants while they were continually heat-induced, with a remarkable production between 24 h and 32 h of sustained heat to reach a maximum of 1 µg/mg DW for the Ara2-4 n°4 lines (Figure 4 and Figure S3). Lines descending from Ara2-4 n°13 yielded a maximum of 0.8 µg/mg DW after a 32 h induction at 40 °C, while those coming from n°8 produced very low to undetectable amounts (Figure 4). A trace amount of casbene was detected in unheated plants, and interestingly, slightly more casbene was detected in unheated but unwatered plants, showing that drought can modestly trigger induction (Figure 4). It may be the combination of heat and accelerated drought that allowed such accumulation of casbene.

**Figure 5:**
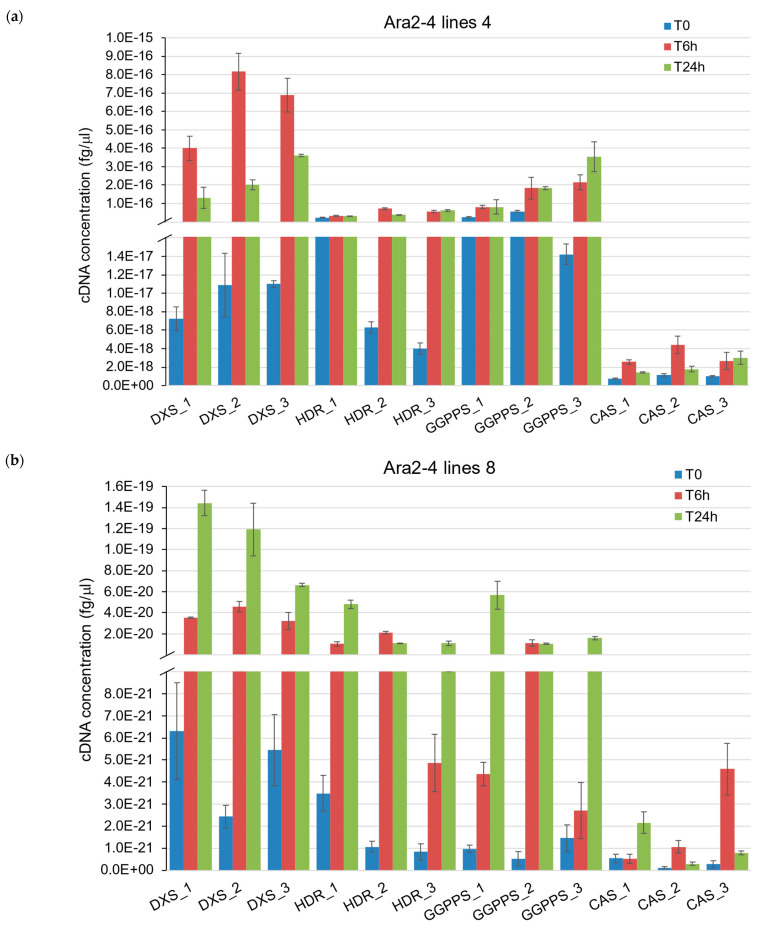

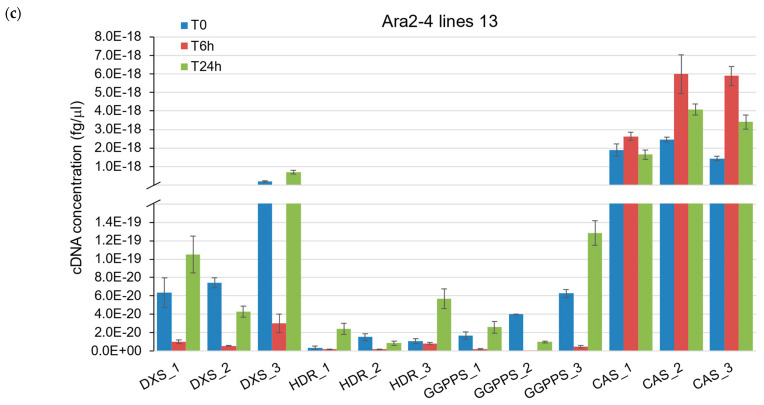
RT-qPCR on each transgene in the inducible lines of the T3 generation. (**a**) Lines from Ara2-4 n°4. (**b**) Lines from Ara2-4 n°8. (**c**) Lines from Ara2-4 n°13. Three plants from each transgenic line were used.

To confirm the successful expression of all the genes involved in the biosynthesis of casbene in these lines, we assessed their transcription levels. This does not directly indicate the levels of the corresponding enzymes produced, but it is a necessary step as the sole detection of casbene is not sufficient to confirm complete pathway activation. For each line, we utilized three plants to extract the RNA, convert to cDNA, and perform a real-time quantitative PCR (RT-qPCR). We measured the level of gene expression of non-induced plants and on plants continuously heated for 6 h and 24 h. As the genes *AtDXS*, *AtHDR*, *AtGGPPS*, and *JcCAS* are not present in *N. benthamiana* WT, we used the Ara2-4 plasmid containing the four cloned target sequences to perform a standard curve before the qPCR (Figure S4). We then used the linear equations of each standard curve to extrapolate the expression of our genes of interest in the transgenic lines (Figure 5).

While our initial aim was to demonstrate the expression of all genes in the transgenic lines, we observed a trend of varying expression levels, with some genes exhibiting expression levels that were orders of magnitude higher or lower than others, but with no consistent pattern observed. For instance, *AtDXS* transcripts in the n°4 lines were 200 times more abundant than those of *JcCAS* at T6h (Figure 5a), while in the n°13 lines, the opposite was true (Figure 5c). Notably, *JcCAS* expression was in the same order of magnitude in both n°4 and n°13 lines, with cDNA detection ranging between 4 × 10^−18^ and 6 × 10^−18^ fg/mL after 6 h of induction (Figure 5a,c). The excess level of precursor gene transcripts in the n°4 lines may contribute to a higher yield of casbene after induction, in contrast to the n°13 lines. In the Ara2-4 n°13 lines, *JcCAS* appears to peak transcriptionally after 6 h of induction, while *AtDXS*, *AtHDR*, and *AtGGPPS* transcripts are repressed compared to T0 and T24h. The Ara2-4 n°8 lines exhibited a lower expression of *AtDXS*, *AtHDR*, *AtGGPPS*, and *JcCAS* compared to lines n°4 and n°13, particularly for *JcCAS*, which was 1000 times lower (Figure 5b) and may not allow for the accumulation of casbene beyond detectable levels (Figure 4). 

## 3. Discussion

In this work, we demonstrated that we can update the utility of heat-inducible promoters for practical applications such as metabolite production. We created a robust chimeric promoter called Ara2 by fusing the heat-shock elements of *Arabidopsis* P-Hsp101 with the −1 to −89 bp sequence of the 35S promoter and the 5′ UTR leader of TMV. The high versatility of combining core promoters with *cis*-regulatory elements means that a countless number of variations have been and could have been tested [44]. Through transient expression assays, we observed that previously characterized promoters yielded inferior results, possibly due to incompatibility with *N. benthamiana*’s endogenous transcription factors or suboptimal heat-shock conditions. For instance, P-Hsp18.2 functions optimally in BY2 *Nicotiana tabacum* cells when exposed to 37 °C for 2 h [45], while P-Hsp70B functions perfectly in *Arabidopsis thaliana* after 30 min exposure at 40 °C [36]. Although the Ara2 promoter functioned particularly well in *N. benthamiana*, this does not guarantee similar performance in other plant species. Our primary objective was to obtain a short and functional promoter to enhance the expression of genes encoding key enzymes for metabolite production specifically in *N. benthamiana* and not to optimize expression with a reporter gene. Heat stress is characterized by an increase in temperature between 10 °C and 15 °C above the normal growing temperatures [46], thus we used heat-shock conditions that would constitute a real stress for this plant native to hot regions of Australia [47]. However, it could be interesting to develop cold-inducible promoters if the target metabolites are heat sensitive [48].

Furthermore, we linked the Ara2 chimeric promoter to three precursor supply genes *DXS*, *HDR*, and *GGPPS* with the casbene synthase *CAS* to synthesize casbene in *Nicotiana benthamiana*, a diterpene not naturally present in this species. These genes were assembled into a single vector with a fifth gene conferring basta resistance, all contained within a relatively large 11 kb multigenic T-DNA. This is still far from the 25 kb threshold, which may become difficult to integrate into the genome, especially if we use non-BIBAC vectors [49]. We successfully integrated the 5-gene cassette in *N. benthamiana*, then clearly showed expression of the four biochemical pathway genes under inducible promoter control. The transgene induction is presumably due to *N. benthamiana* heat responsive transcription factor(s) binding to *cis* elements on the synthetic promoter designs described herein, with those heat shock elements contained within the HSP101 promoter being most reliable for our purposes.

More significantly, we detected casbene in most of the transgenic plants after heat induction. Our data showed that a continuous heat induction of 40 °C for 32 h allowed the accumulation of casbene at up to 1 mg/mg DW in some transgenic lines. We also demonstrated that all transgenic lines display transcription of the introduced genes, despite variability in their expression levels. The variability in expression may be attributed to potential positional effects on gene transcription due to integration at different locations in the genome. Furthermore, the uniformity of promoter sequences controlling the four genes can lead to transcriptional silencing, as previously observed [50], which may account for the variability in gene expression.

In our study, we observed consistent leaks of the Ara2 promoter in GFP transient expression assays, casbene production experiments, and RT-qPCR data. Interestingly, while the leaks were evident in the RT-qPCR results, they did not correlate with the amount of casbene detected. This discrepancy highlights the complexity of gene regulation and suggests that factors other than promoter leaks might influence the final production of target proteins or metabolites. Nonetheless, our findings underscore the importance of developing strictly non-leaky promoters for future research in this area. Particularly when dealing with proteins and/or metabolites that are toxic at very low levels, the use of non-leaky promoters during normal growth stages becomes crucial to prevent undesirable effects. Developing such promoters represents the next challenge in harnessing inducible systems for precise control of gene expression in plants.

## 4. Materials and Methods

### 4.1. Cloning of GFP Constructs and Transient Expression of GFP Driven by Heat Inducible Promoters in N. benthamiana

Chimeric DNA sequences containing the heat-shock elements of native promoters and the different regions of the CaMV35S promoter were synthesized by our partners at GlaxoSmithKline Pharmaceuticals (GSK). These sequences had the right overhangs to be directly cloned into level 0 acceptor vector for promoter only provided by the MoClo Toolkit (Addgene) [38,39] using the long protocol described by the manufacturer. All components and enzymes used for the cloning, T4 ligase (400 U/mL), BsaI (10 U/mL), BpiI (10 U/mL), and BSA (10×) were purchased at ThermoFisher (Waltham, MA, USA). The 5′UTR TMV was obtained from a separate vector provided by the MoClo Plant Parts kit (Addgene, Watertown, Massachussets USA) (Table S1) [33]. To reconstitute the native P- Hsp101, the synthetic sequences without the 35S part were re-amplified using HiFi polymerase (PCR Biosystems, London, UK) and cloned into acceptor vector level 0 for promoter only (Tables S1 and S2). The gBlock™ (Integrated DNA Technology, Coralville, IA, USA) sequence of the 5′UTR was ordered and cloned into the MoClo acceptor level 0 for 5′UTR. The short P-GmHsp17.3B containing the heat shock elements and the 5′UTR was ordered directly as a gBlock™ and cloned into the acceptor vector level 0 for promoter + 5′UTR. P-Hsp18.2 and P-Hsp70B were purchased on Addgene from the GoldenBraid kit [37]. Both promoters were re-amplified with HiFi polymerase to add the BpiI overhangs and cloned into level 0 acceptor vector for promoter + 5′UTR. P-Hsp70-B had to be re-amplified by removing an internal BpiI site (Table S2).

We then combined in level 1 acceptor vectors the synthetic or re-amplified promoter sequences: the native or TMV 5′UTRs when relevant, the CDS of *Turbo GFP* (*tGFP*), and the terminator of the octopine synthase (OCS). These single transcription units in the level 1 vectors were further cloned into the level 2 vector, the final plasmid intended for transient expression. Level 2 vectors were transformed into *Agrobacterium tumefaciens* LBA4404 using the freeze-thaw method [51]. 

Transient expression in *Nicotiana benthamiana* was performed by dipping the plants in an infiltration medium containing 10 mM MgCl_2_, 200 mM acetosyringone, 0.015% Silwet L-77, and each *Agrobacterium tumefaciens* strain at a final OD_600_ of 0.2 [12]. We performed co-infiltration by mixing our constructs with another *Agrobacterium* strain containing an empty pEAQ-HT vector [52] containing the suppressor of silencing *P19* [53] donated by Professor Lomonosoff. This pEAQ-HT vector was exclusively used for co-expression of the P19 in the transient expression assays and was not employed in any other experiments or in the design of the synthetic promoter. Three plants per construct were infiltrated and subsequently subjected, or not, to heat-shock.

### 4.2. Heat-Shock and Visualization of GFP in Nicotiana benthamiana

Plants were grown in individual pots with F2 + S seed and modular compost (Levington Advance, Cardiff, UK) and cultivated in a growth room equipped with white fluorescent lamps and set at 22 °C during the day (16 h) and 20 °C during the night (8 h). Four days following infiltration, the triplicate of plants for each construct were heated for 2 h at 40 °C in a Sanyo plant growth cabinet featuring the same light regime as the growth room. After timing the end of the heat-shock with the end of the 16 h day light period, the plants were transferred back to the growth room to collect the material at the different time-points. The same trio of plants were visualized under UV at the different time-points, to ensure a direct comparison of fluorescence patterns and eliminate potential variations arising from different plant individuals. 

The *tGFP* used in this study was codon-optimized for plants [33] and came from the arthropod *Pontellina plumata*, which has maximum excitation and emission wavelengths at 482 and 502 nm respectively [54]. We used a blue-light transilluminator SafeVIEW-Mini2 (Cleaver Scientific, Rugby, UK) emitting a 470 nm blue light to visualize the GFP in the leaves. We captured the fluorescence by using an 18–55 mm lens and a DSLR Canon camera EOS 1100D attached to a horizontal adjustable camera holder Kaiser 5411 (Kaiser, Schalksmühle, Germany). Manual settings of the camera were as follows: auto white balance (AWB), ISO 800, aperture f/4.5, focal length 32 mm, and exposition 1 s. The photos were cropped uniformly with the Gimp software 2.10.32 to obtain 1.7 × 1.7 cm pictures. They were also processed to amend the color balance. By putting the original temperature at 9300 K and the intended temperature at 1850 K, it was possible to reveal the green color in the fluorescent leaves or to increase the red color in the non-fluorescent leaves (Figure S1 for the original pictures). This especially allowed for confirmation of whether or not fluorescence was visible in the non-heat-induced plants.

### 4.3. Stable Expression in Nicotiana benthamiana of the Ara2-4 Cassette and Heating Treatment

*Arabidopsis thaliana* cDNA of *DXS* (AT4G15560.1) and *GGPPS11* (AT4G36810.1) [55] coding for plastidial enzymes referenced in TAIR (The *Arabidopsis* Information Resource) were cloned into MoClo level −1 vectors [38,39] to remove the endogenous BbsI or BsaI resctriction sites. *HDR* (AT4G34350.1) [56] and cDNA of *CAS* from *Jatropha curcas* [15] were cloned directly into MoClo level 0 vectors (Tables S1 and S2). These coding sequences were then cloned into the level 1 vector with the elements constituting the Ara2 promoter and different terminators provided by the MoClo Plant Part Kit [33]. Together with the gene coding for *Bar* (bialaphos/glufosinate/Basta resistance cassette), also available in the MoClo kit, the transcription units were assembled in the level 2 vector intended for stable transformation. 

This level 2 construct was transformed into *Nicotiana benthamiana* using the *Agrobacterium tumefaciens* leaf disc method [57]. Transformation and Basta selection for the different generations of transformants was as described in Forestier et al., 2021. Briefly, six-week-old *N. benthamiana* leaves were sterilized and cut into 1 cm diameter discs. The discs were soaked in a co-cultivation solution consisting of 4.3 g/L of MS medium M0221 (Duchefa, Haarlem, The Netherlands), 30 g/L of anhydrous glucose, 100 mg/L of myo-inositol, 0.5 mg/L of the vitamins nicotinic acid, thiamine-HCl, and pyridoxine, 2 mg/L of glycine, and adjusted to pH 5.7–5.8 with a few drops of KOH. *A. tumefaciens* LBA4404 containing the vectors of interest was prepared and added to the co-cultivation solution. Infected leaf discs were incubated on co-cultivated for 3–4 days on a medium with 0.1 mg/L of 1-naphthaleneacetic acid (NAA) and 1 mg/L of 6-benzylaminopurine (BAP) (Sigma-Aldrich, Burlington, MA, USA), then transferred to selection medium with 5 mg/L of glufosinate and 500 mg/L of cefotaxime (Sigma-Aldrich). Shoots appeared after 30–40 days and were transferred to rooting medium (2.65 g/L of modified MS n°4 M0238 from Duchefa, 825 mg/L of NH_4_NO_3_, 30 g/L of sucrose, 100 mg/L of myo-inositol, 0.5 mg/L of the same 3 vitamins as the co-cultivation medium, KOH to adjust the pH to 5.7–5.8, and 6 g/L of agar) with doubled concentration of glufosinate. Roots were developed 15–30 days after transfer, allowing the transformed seedlings to be put into the same compost used for WT plants. 

Seeds of the subsequent generations were vapor-phase sterilized and sown on a germination medium (4.4 g/L MS medium M0221 from Duchefa, 10 g/L sucrose, 100 mg/L myo-inositol, 0.5 mg/L nicotinic acid and pyridoxine, 1 mg/L thiamine, a few drops of KOH to adjust to pH 5.7–5.8, and 6 g/L agar) containing 10 mg/L glufosinate to perform segregation tests and estimate the copy number of each transgene. Because methods such as RT-qPCR demonstrated limited reliability for determining the precise copy number in *Nicotiana benthamiana*, we employed segregation ratios for the Basta selectable marker genes in the T1 and T2 progenies of independent transformants to determine the copy number and zygosity of the transgenes. A 3:1 ratio of resistant to sensitive plants in the T1 generation indicated a single copy of the transgene, while a 100% resistance rate in the T2 generation suggested homozygosity.

For heat-induction of the T3 progenies and WT at 40 °C, three plants from each lineage and for each heat exposure were transferred to the same Sanyo growth cabinet described above (except the non-induced plants). Heating was initiated at 8 am in order to collect the plant material at convenient times for the different time-points. Watering was stopped 24 h before induction for the heated plants and the “no induction–no water” ones.

### 4.4. Isolation and Quantification of Casbene in the Transgenic Lines

To detect and quantify the production of casbene in heat induced and non-heat-induced transgenic lines, three plants were extracted for each condition. We took around 200 mg of freeze-dried material, ground it for 30 s with a steel bead at 30 Hz in a Retsch homogenizer (Retsch^®^, Haan, Germany), and extracted it with 1 mL of ethyl acetate containing 100 µg/mL of β-caryophyllene. The samples were shaken overnight at 2000 rpm on a Vibrax^®^ VXR basic shaker (IKA, Staufen, Germany), then centrifuged, and 100 µL amounts of the extracts were directly used for GC-MS analysis. The GC oven was fitted with a Restek RTX-5SIL MS capillary column (30 m, 0.25-mmID, 0.25 mm df). The oven temperature was set at 100 °C for 2 min and then increased to 300 °C at a rate of 5 °C min^−1^. Mass spectral data were acquired over the *m*/*z* range of 50 to 450 in positive electron ionization mode at −70 eV. Casbene was quantified by determination of the total ion chromatogram (TIC) peak area and comparison to the peak area of the internal standard, β-caryophyllene. 

To assess the effect of different heat-induction durations on casbene production in the T3 progenies of each selected independent transformant, we performed a one-way ANOVA test followed by Tukey’s multiple comparisons test to compare the means of casbene production among the different heat exposures, with three plants analyzed per heat exposure and for each independent transformant. All statistical analyses were performed using GraphPad Prism version 9 for MacOS (GraphPad Software, San Diego, CA, USA, www.graphpad.com, last accessed on 23 June 2023).

### 4.5. RNA Isolation and Real-Time Quantitative PCR

Total RNA of three transgenic plants coming from the transgenic lines Ara2-4 n°4, n°8, and n°13 were extracted before heat induction and after 6 h and 24 h of continuous heat at 40 °C using the RNeasy kit from Qiagen. Total RNA was also isolated from three untreated untransformed *N. benthamiana* plants as control samples. Harvested leaf tissues were frozen into liquid nitrogen, disrupted with a TissueLyser II (Qiagen, Hilden, Germany), and extracted with the buffer RLC described in the manufacturer protocol. Reverse transcription of RNA was performed at 55 °C for 60 min with the Superscript III enzyme and the Oligo d(T)_20_ primers (Invitrogen, Waltham, MA, USA). We included the RNase H (ThermoFisher, Waltham, MA, USA) step after the reverse transcription as suggested by the manufacturer. 

The resulting 30 cDNA samples were used to conduct real-time quantitative PCR on transcripts of *AtDXS, AtHDR, AtGGPPS*, and *JcCAS*. PCR primers used are detailed in Table S2. Expression levels of a house-keeping *ACTIN* gene were monitored to check that qPCR reactions were uniform in all samples. A Roche LightCycler^®^ 480 instrument using the Sybr Green Master I (Roche, Rotkreuz, Switzerland) was used to conduct the qPCR. Cycling conditions can be found in Table S3.

To obtain standard curves for *AtDXS, AtHDR, AtGGPPS*, and *JcCAS* genes, qPCR of the 4 transgenes present in the Ara2-4 plasmid was conducted on a 10-fold dilution series from 1 ng/mL to 0.1 pg/mL. Standard curves were acquired by plotting the Ct values against the decimal logarithm of plasmid DNA concentrations.

## Data Availability

Data supporting the reported results can be made available upon request to the corresponding author.

## Supplementary Materials

The following supporting information can be downloaded at: https://www.mdpi.com/article/10.3390/ijms241411425/s1.
