# Supplementary material for: An Engineered Heat-Inducible Expression System for the Production of Casbene in Nicotiana benthamiana
Source: Int J Mol Sci. 2023 Jul 13;24(14):11425. doi: 10.3390/ijms241411425 (PMC10379985; doi:10.3390/ijms241411425)
Supplement: Supplementary file 1 [file ijms-24-11425-s001.zip › Edited_supplementary figures.pdf]

**Figure S1. Original pictures of figure 2 showing GFP expression driven by chimeric and original heat-shock promoters.**

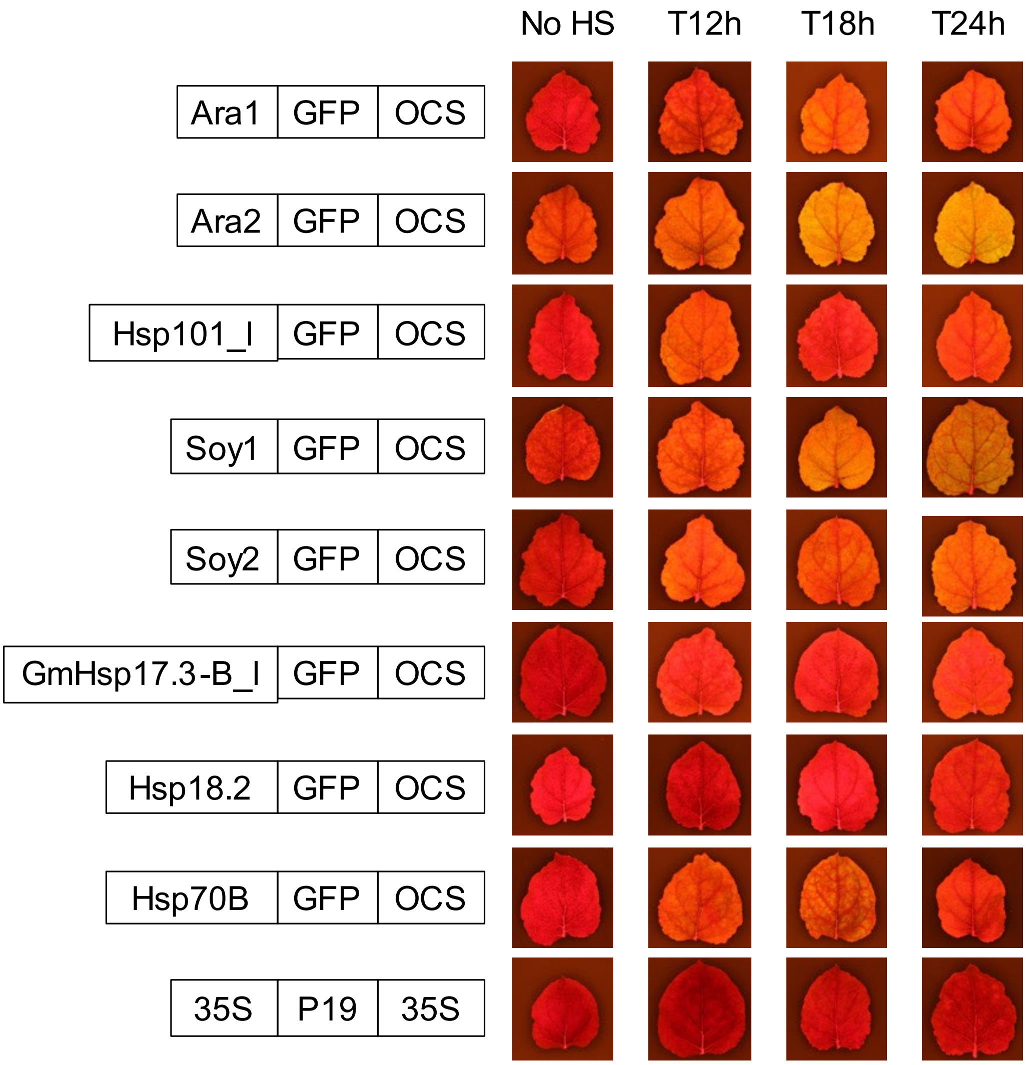

**Figure S2. General aspect of *Nicotiana benthamiana* wild-type and T3 transgenic lines at 5 weeks-old**. (a) WT. (b) Ara2-4 n°4. (c) Ara2-4 n°8. (d) Ara2-4 n°13.

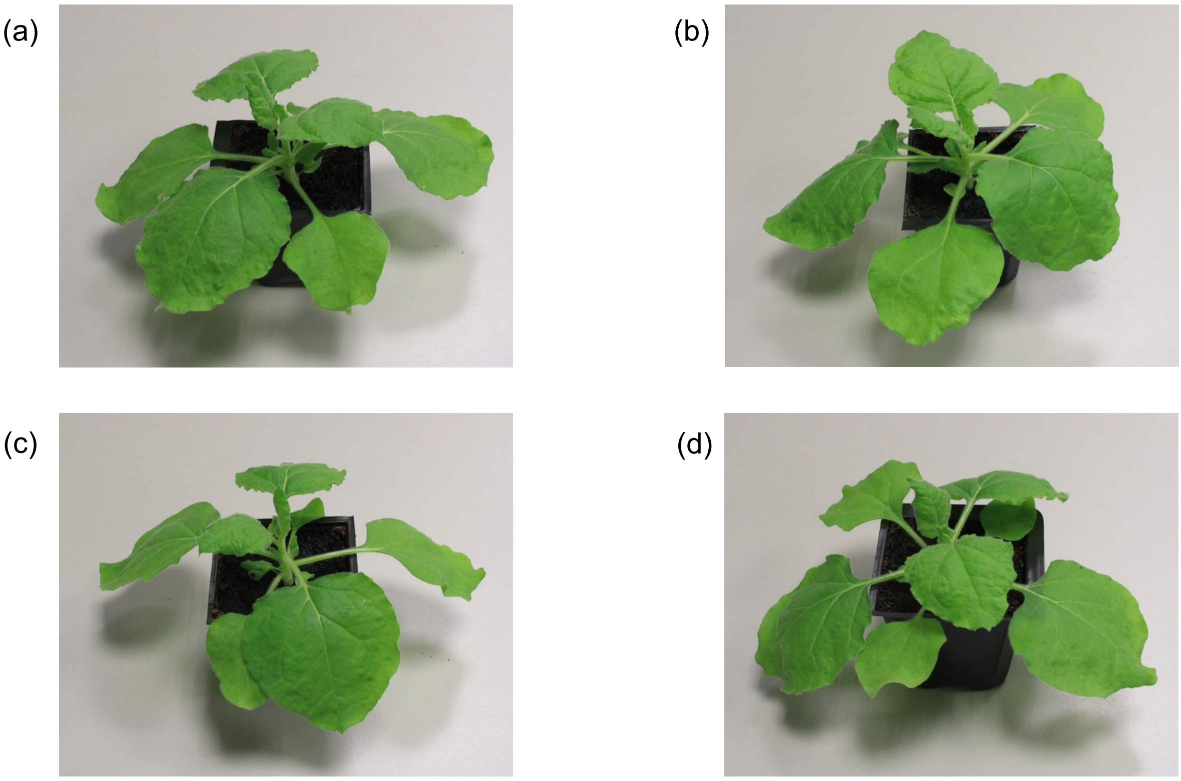


**Figure S3. GC-MS chromatograms showing production of casbene in a heat-inducible line.** (a) Ethyl acetate extract of WT *Nicotiana benthamiana* heated for 32h. (b) Ethyl acetate extract of T3 line Ara2-4 n°4 heated for 32h. 1, internal standard β-caryophyllene. 2, casbene.

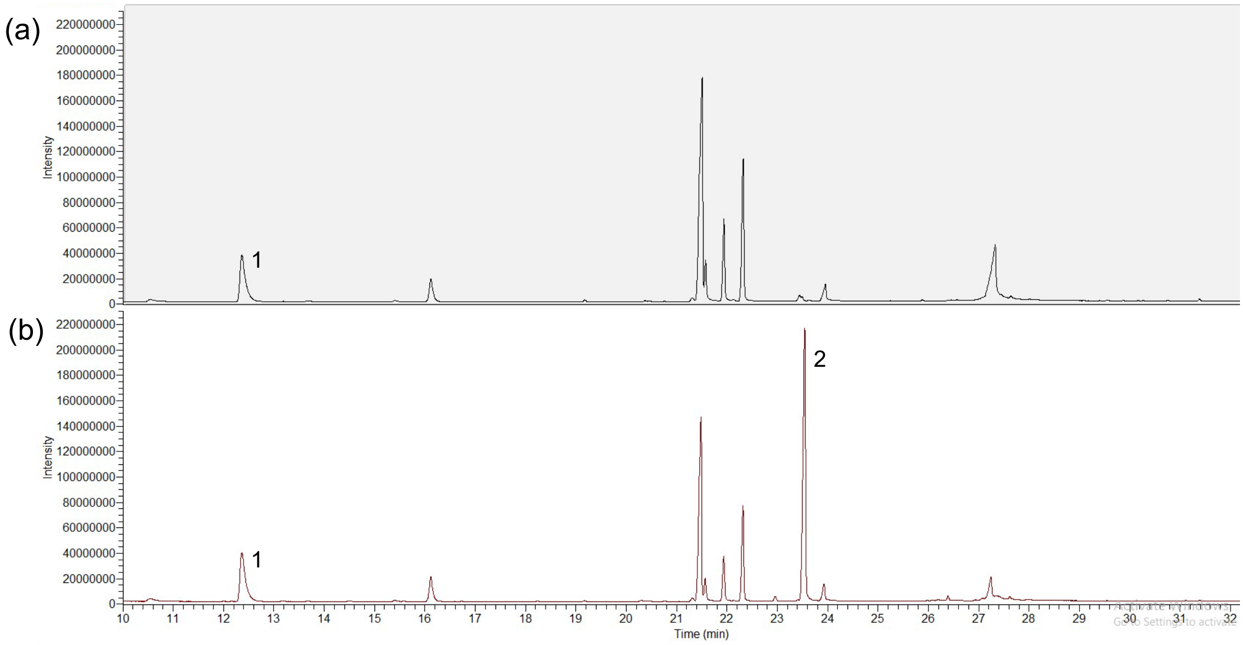

**Figure S4. Standard curve of the transgenes *DXS*, *HDR*, *GGPPS* and *CAS* present in the Ara2-4 vector.** Standard curves of the four cloned genes in the Ara2-4 plasmid (a) and PCR efficiency (b).

(a)

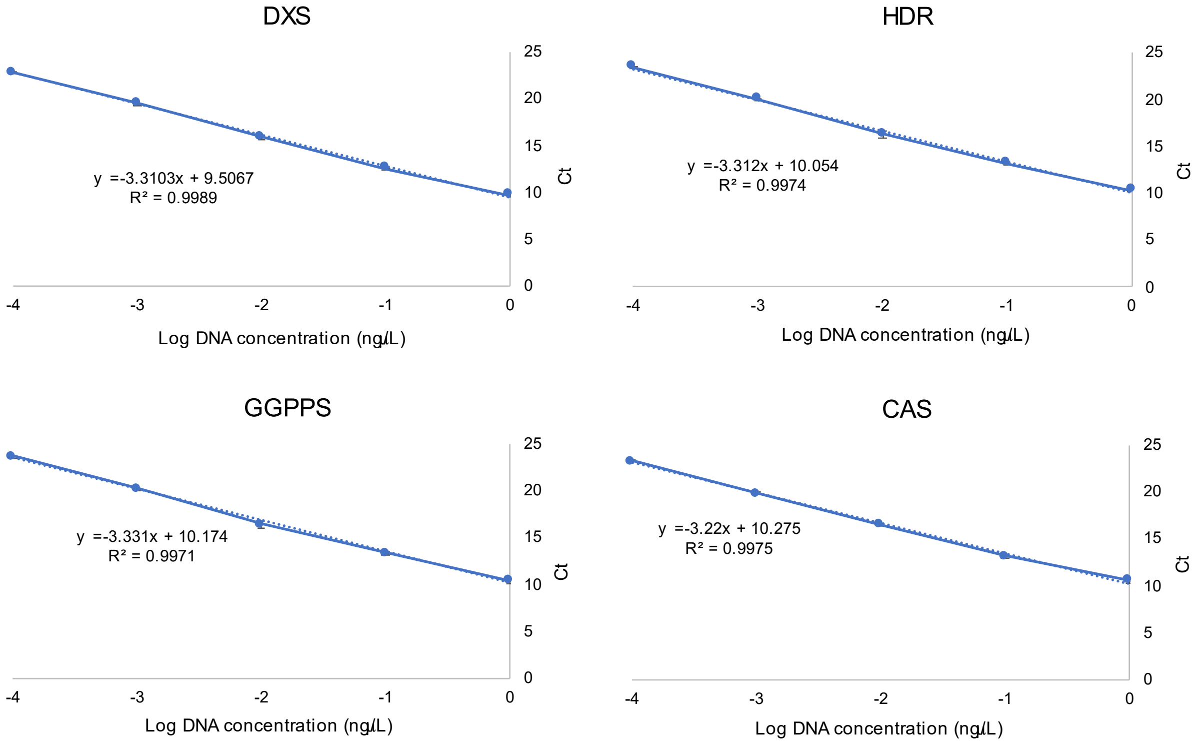


(b)

| | Efficiency | % efficiency |
|---|---|---|
| DXS | 2.005 | 100.5 |
| HDR | 2.004 | 100.4 |
| GGPPS | 1.996 | 99.6 |
| CAS | 2.044 | 104.4 |
